# Improving Accuracy in Graphite Furnace Atomic Absorption Spectrometry Through Peak Shape Monitoring

**DOI:** 10.6028/jres.093.121

**Published:** 1988-06-01

**Authors:** Markus Michaelis, Wolfhard Wegscheider, Hugo M. Ortner

**Affiliations:** Institute for Analytical Chemistry, Mikro- and Radiochemistry, Technical University Graz, A-8010 Graz/ Australia; Metallwerk Plansee GmbH, A-6600 Graz/ Australia

Due to the good detection limits of graphite furnace atomic absorption spectrometry (GFAAS), situations frequently arise when an analyte is determined in the presence of a large excess of concomitants. It is in these cases when the improvements made in furnace design, heating rate optimization, Zeeman background correction, platform atomization and application of matrix modifiers are still not sufficient to provide the analytical chemist with reliable data.

On the other hand, large gains in mechanistic insight have not yet yielded a model that can successfully cope with atomization characteristics in samples with a large matrix/analyte ratio. Typical accounts of the current state are found in [1] for explicit differential equations formulations and in [2] for the Monte Carlo approach, but there is no real difference in the predictive abilities of the two. Nevertheless, it is clear that a wide variation of peak shapes are observed and qualitatively monitored by everyone practicing GFAAS.

In this work we describe a characterization of peak shapes by various parameters, such as appearance time, mean, mode, peak width, area/height, skewness and kurtosis. This is done by attempting to alter the peak shape systematically through deliberate addition of concomitant elements in a fractional factorial experiment [3,4]. The observed shape parameters are then linked to the characteristic mass after discarding those that vary with analyte mass.

## Experimental

A Perkin-Elmer 5000 with HGA 500 and AS 40 autosampler was used throughout. The computations were run on a Perkin-Elmer 7500 data station and on an IBM PC-XT. All data given are Zeeman background corrected and all samples are atomized from a platform. Two different platforms were used. One was the vendor-supplied pyrolytic graphite platform, and the other one was produced from the same type by physical vapor deposition of TaC [5]. A general account of the analytical properties of this TaC-coated platform for ETAAS can be found elsewhere [6]. All reagents were of analytical grade. Doubly distilled water and subboiled nitric acid were employed.

Sn, Se, and Rh served as model analytes to study the behavior of elements different in both chemistry and volatility. Ten micrograms of each palladium and magnesium as nitrates were used as matrix modifiers for Se, and 200 μg ammonium dihydrogen phosphate was used for Sn. First, temperature programs were optimized for standard solutions and not altered for any of the subsequently described experiments. Generally, atomization temperatures from the TaC-coated platform tended to be higher than for the ordinary total pyrolytic graphite platform [5].

Calibration curves were run to establish the linear range, but—more importantly—the dependence of peak shape parameters on analyte mass. From this the concentration levels were chosen for the interference experiments to be well within the linear range. Cr, Fe, Na, Zn, Mg and A1 were chosen as potential interferents and added as nitrates in a 29000 (Sn) to 40000 (Se) fold molar excess over the analyte. A fractional factorial design in these interferents was set up which was replicated once, giving a total of 64 profiles [4]. These were recorded in random order with 2 atomizations of the standard solution and 2 atomizations of a blank solution before and after the run of 64.

## Results and Discussion

Peak shape parameters can serve as a reduced description of the entire atomization curve and should be defined to carry information on the (largely) unknown fundamental mechanisms in GFAAS. In this work it was assumed that—if not the mechanisms—at least kinetic and thermodynamic parameters governing the processes would be altered by the presence of interferents and result in changes of peak shape. Consequently, the observed atomization curve could be regarded as a faithful representation of the free atom population in the detection volume and should be accessible to a nonparametric description. The peak shape parameters used in this study are given in table 1. As these parameters were chosen to model sensitivity in unknown samples, it is required that they be fairly insensitive to analyte mass. This could be shown to be the case for all, but the parameter “length” [7] which was therefore not used in further studies.

**Figure 1:**
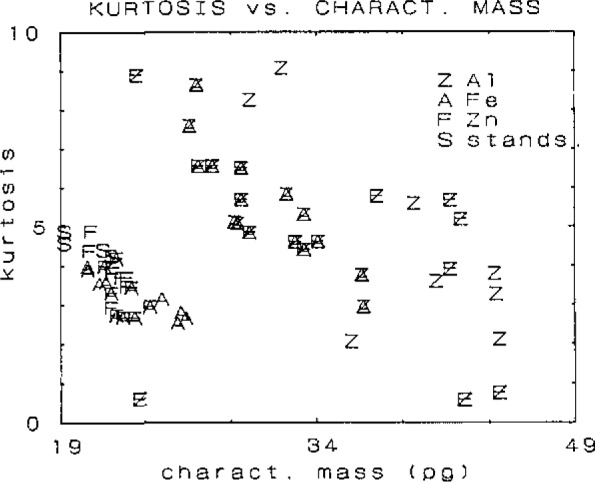
Variation of kurtosis with nature of analyte and change of characteristic mass.

Figure 1 shows the change in kurtosis with the type of interferent and with the resulting characteristic mass. Of course, in a real analytical setting situations may arise where the interferent is not identifiable. It is therefore necessary that the characteristic mass is modelled independent of the nature and mass of an interferent. This can be attempted using only the peak shape parameters.

The technique employed for this modelling is known in the literature as partial least squares (PLS) regression [8]. The data from the interference study, supplemented by the four data on the standards were subjected to PLS. A subset of the data was randomly removed for the modelling step and used to assess the predictive ability afterwards. Table 2 gives an overview of the results. The optimal number of dimensions for the PLS model varied between 2 and 6 with 62 profiles fitted; six atomization curves were used for predictions. The relative importance of the different peak shape parameters is different for each data set. Detailed results on this subject will be published elsewhere [9].

The success of these initial experiments is obvious. In practice the applicability of the proposed scheme will depend (i) on whether the majority of changes in peak shape can be observed in the initial experimental design, and (ii) on the validity of the implicit assumption that most interferences manifest themselves by changes of peak shape. Both questions cannot be answered without full insight into the mechanisms of atomization. In the meanwhile, the approach can serve as a means to warn the analyst about the unexpected occurrence of interferences. The model for characteristic mass can serve to improve accuracy. This by itself should help to make data from ETAAS more reliable.

## Figures and Tables

**Table 1:** Peak shape parameters

Name	Description
area/height	(self explanatory)
appearance time	time when signal rises significantly above baseline noise
length	time interval from appearance time to drop of the signal below significance
standard deviation	width of the peak measured by assuming the signal to be the probability density curve of the free atom population in the analysis volume
mode	time of peak maximum
mean	arithmetic mean time of the peak
skewness	measure of asymmetry of peak [3]
kurtosis	measure of slimness of peak [3]
baseline noise	absorbance corresponding to the average spread of baseline noise

**Table 2:** Results of modelling characteristic mass from peak shape parameters

Element	Platform	Rel. residuals %	Rel. prediction error %
Se	pyro	2.0	11
	TaC-coated	2.4	4.7
Rh	pyro	2.8	4.5
	TaC-coated	8.6	10.6
Sn	TaC-coated	0.6	8.6
